# Melatonin and Its Effects on Plant Systems

**DOI:** 10.3390/molecules23092352

**Published:** 2018-09-14

**Authors:** Rahat Sharif, Chen Xie, Haiqiang Zhang, Marino B. Arnao, Muhammad Ali, Qasid Ali, Izhar Muhammad, Abdullah Shalmani, Muhammad Azher Nawaz, Peng Chen, Yuhong Li

**Affiliations:** 1 College of Horticulture, Northwest A&F University, Yangling 712100, China; rahatsharif2016@nwafu.edu.cn (R.S.); yyxyxc180108@nwafu.edu.cn (C.X.); zhanghaiqiang@nwafu.edu.cn (H.Z.); alinhorti@yahoo.com (M.A.); 2 Department of Plant Biology (Plant Physiology), Faculty of Biology, University of Murcia, Campus de Espinardo, 30100 Murcia, Spain; marino@um.es; 3 Department of Horticulture, Faculty of Agriculture, Akdeniz University, 07059 Antalya, Turkey; qasidmrz01@gmail.com; 4 State Key Laboratory of Crop Stress Biology in Arid Areas, College of Life Sciences, Northwest A&F University, Yangling 712100, China; izeyaar@gmail.com (I.M.); abdullqadir36@yahoo.com (A.S.); 5 Department of Horticulture, University college of Agriculture, University of Sargodha, Sargodha 40100, Pakistan; azher490@hotmail.com; 6 College of Life Science, Northwest A&F University, Yangling 712100, China

**Keywords:** melatonin, abiotic stress, biotic stress, antioxidants, gene expression, postharvest, mitochondria

## Abstract

Melatonin (*N*-acetyl-5-methoxytryptamine) is a nontoxic biological molecule produced in a pineal gland of animals and different tissues of plants. It is an important secondary messenger molecule, playing a vital role in coping with various abiotic and biotic stresses. Melatonin serves as an antioxidant in postharvest technology and enhances the postharvest life of fruits and vegetables. The application of exogenous melatonin alleviated reactive oxygen species and cell damage induced by abiotic and biotic stresses by means of repairing mitochondria. Additionally, the regulation of stress-specific genes and the activation of pathogenesis-related protein and antioxidant enzymes genes under biotic and abiotic stress makes it a more versatile molecule. Besides that, the crosstalk with other phytohormones makes inroads to utilize melatonin against non-testified stress conditions, such as viruses and nematodes. Furthermore, different strategies have been discussed to induce endogenous melatonin activity in order to sustain a plant system. Our review highlighted the diverse roles of melatonin in a plant system, which could be useful in enhancing the environmental friendly crop production and ensure food safety.

## 1. Introduction

Melatonin was discovered in the bovine pineal gland and called as vertebrate pineal secretory molecule [1,2]. The pineal gland is an organ present in animals’ bodies, responsible for the production of melatonin in order to control the behavior of the body toward changing photoperiod and also serve as a neuronal protective antioxidant [3]. However, in plants, the existence of melatonin has been reported in more than 20 dicotyledonous and monocotyledonous plant families [4,5]. The name melatonin was given to this biomolecule after Lerner et al. [6] stated that an indole molecule is responsible for causing skin lighting in the frog. In addition to that, it is the most important factor for controlling the circadian cycle in different vertebrates; the secretion of melatonin reaches to the highest level during the night time, which makes it the peak signaling molecule of darkness [2]. Moreover, melatonin is an important antioxidant that can be taken in diet and also the body produces it endogenously, though its production degrades gradually with increasing age [2,7,8]. In plants, the role of melatonin has been extensively studied [9,10,11]. 

Since the discovery of melatonin in 1965, about 34,000 research materials regarding melatonin are available on Scopus database; this highlights the importance of this molecule in that it has been studied extensively. Because of its significant effects on plant systems, it attracts scientists and young researchers from the diverse field of plant sciences [12,13,14,15]. It is considered as a central indoleamine neurotransmitter, largely involved in the diverse biological process and accepted as an important plant metabolite [16,17]. Additionally, melatonin has been reported for its involvement in improving seed germination, fruit ripening, photosynthesis, biomass production, circadian rhythm, redox network, membrane integrity, root development, leaf senescence, osmoregulation, abiotic stress (salt, drought, cold, heat, oxidative, heavy metals) [18,19,20,21,22,23,24,25,26]. Moreover, it has been reported to play a beneficial role in the protection of plants against biotic stresses [14]. Furthermore, melatonin induces gene expression which helps the plant to cope with biotic and abiotic stresses [27]. Therefore, it could be of great importance to utilize melatonin as a bio-stimulator for sustainable crop production without affecting the external environment.

**Figure 1:**
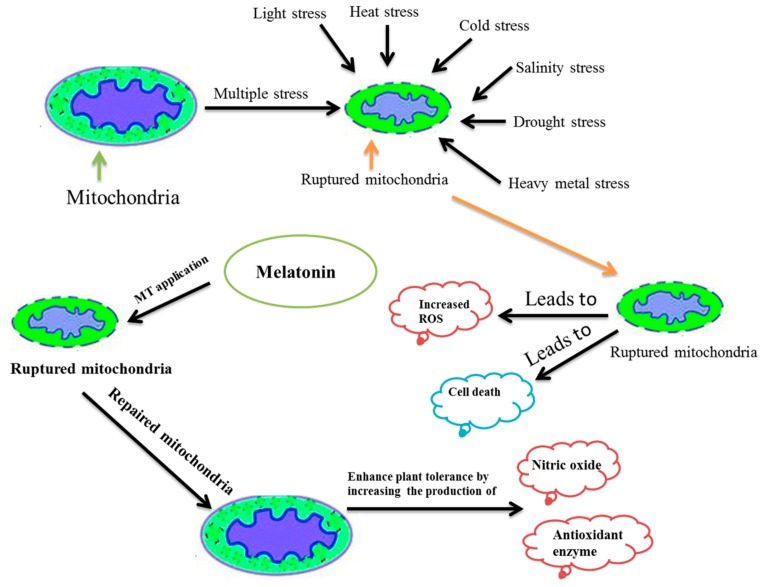
Schematic representation of melatonin defense mechanism pathway.

The purpose of this review is to highlight the various aspects of melatonin from the plethora of research available over its role in protecting plants from abiotic, biotic and post-harvest stresses. Additionally, the contribution of melatonin in regulating gene expression has been presented here. The melatonin defense mechanism has also been presented in this review with illustration and diagrammatic sketch (Figure 1). Furthermore, we discussed the melatonin concentration in different plants as well as the strategies to improve the endogenous melatonin content to restore the defense in plants.

## 2. The Occurrence of Melatonin in Plants

Melatonin is an ancient molecule derived from cyanobacteria which was introduced to animals and plants through evolution [28]. Melatonin is an indolic compound derived from tryptophan and it was found during 1995 in plants. It is found in almost all the plant species, varying in amount according to the plant tissues in light depending manner [29,30]. Though the level of melatonin is higher in aromatic plants and leaves than that of seeds [29]. In higher plants, melatonin was firstly discovered in 1993 in the Convolvulaceae ivy morning glory (*Pharbitis nil* L., *syn. Ipomoeanil* L.) and in tomato fruits (*Solanum lycopersicum* L.), however, due to some unknown reasons, the results were published two years later in 1995 [31,32]. Moreover, the majority of the plants reported to contain melatonin contents are from family *Rosaceae*, *Vitaceae*, *Poaceae*, *Apiaceae* and *Brassicaceae*, however, plants from other species have also exhibited higher endogenous melatonin contents. During the last decade, several researchers revealed that the concentration of melatonin also differs between the varieties of the same species, depending on location, growth stage, organ and harvest timing [33,34,35]. Endogenous melatonin was reported to play an important role in the regulation of plant growth attributes in various species [36]. For that reason, several detection methods and assays have been reported for the determination of melatonin content in plant samples that includes radioimmunoassay (RIA), enzyme-linked immunosorbent assay (ELISA), gas chromatography-mass spectrometry (GC-MS), and high-performance liquid chromatography (HPLC) with electrochemical detection (HPLC-ECD), fluorescence detection (HPLC-FD), or high-performance liquid chromatography-mass spectrometry HPLC-MS [37]. However, RIA has been reported to be less reliable in detecting melatonin content compared with other methods [13]. Therefore, we enlisted the reported melatonin contents and its detection methods in some of the important agronomic and horticultural plants (Table 1). However, these values could be changed as melatonin production is dependent on the day length and also seasonal changes. For example, its production is low during the daytime and high during the night time [38,39]. Thus, the values stated in Table 1 are uncertain unless the circadian, seasonal and plant age variations are known. We believe this information could be helpful for future research programs regarding the detection of endogenous melatonin level.

## 3. Role of Melatonin in Regulating Plant Growth and Physiology

The primary functions of melatonin are as an antioxidant because it is soluble in water and fats and moves freely across the body to any aqueous section [28,51]. Though it has been reported that melatonin improves the overall growth of plants [42,52]. It enhances the coleoptile length of canary grass, barley and wheat [42]. The melatonin-treated maize seeds resulted in better seed vigor and quality and improved seed storage proteins [53]. According to another report, a coating of soybean seed with melatonin significantly improved the leaf growth, plant height, number of pod plants^−1^ and number of seeds per pod [54]. A similar role of melatonin was also observed in etiolated *Lupinus albus* L., where it was found to be responsible for the promotion of vegetative growth, and regenerations of lateral and adventitious roots [55,56]. While in cucumber plants, an increase in the seedling growth, improvement in the nutrient uptake efficiency and enhancement in nitrogen metabolism were perceived after treatment with melatonin, particularly under salt stress conditions [57]. Moreover, it was reported that melatonin treatment improved photosynthetic activity, enhanced redox homeostasis, regulated root growth and development, and seminal root elongation in barley, wheat, sweet cherry and rice [47,58,59,60,61,62]. The plant hormones such as auxin, ethylene, cytokinin, gibberellins, IAA (indole 3-acetic acid) and brassinosteroids are extensively involved in regulating plant growth and development [63]. The effects of these plant hormones can be regulated with the application of exogenous melatonin application [64]. Among them, IAA shared similarities in structure and functions [42,65]. In line with that, exogenous treatment of melatonin enhances the production of IAA [66]. While on the other hand both melatonin and IAA work in the combined and similar fashion as they were reported for enhancing root morphogenesis [52]. In *brassica juncea* plant, exogenously applied melatonin enhanced the IAA level, which further resulted in better root activity [67]. Whereas it influenced the root organogenesis positively in *Mimosa pudica* L. [68]. Therefore, it is assumed that melatonin influenced signal transduction and also had a role in regulating plant physiological and biological processes. To sum up, melatonin could be considered as a biological plant growth regulator to improve the production capacity of a plant.

## 4. Melatonin Effect on Postharvest Produce

The shelf-life and quality of postharvest produce decline due to the deterioration. For this reason, many treatments have been implemented to maintain the quality and shelf life of postharvest fruits and vegetables [69,70,71,72]. Usually, the produce is stored in a cold environment which induces oxidative stress by elevating the production of ROS; this is the main drawback of cold storage [73]. However, treatment with melatonin alleviates the ROS activity and increases the antioxidant enzymes production [70]. In other cases, the application of exogenous melatonin triggered the endogenous melatonin biosynthetic activity via the antagonistic crosstalk with calcium, preventing the product from postharvest deterioration [74]. Additionally, the postharvest quality of horticultural produce is mainly dependent on the preharvest factors as it cannot be increased after harvesting but can only be maintained [75]. In line with that, the tomato seeds fertigated with melatonin had not only increased their yield but also kept the postharvest quality by exhibiting an increase of vitamin C, lycopene and calcium contents. The treated plants also recorded for more soluble solids and P content than that of control [76]. In another study, the exogenous application of melatonin on the clusters of grapes attached to the vine had altered metabolism of polyphenol, carbohydrate biosynthesis and more importantly ethylene signaling in berries of grapes. The restricted ethylene production resulted in better antioxidant activity [74], which is an important factor for maintaining postharvest quality. Moreover, melatonin regulates salicylic acid, jasmonic acid, nitric oxide and ethylene which collectively generate the resistance against diseases in a very familiar action [64]. The cooperative or antagonistic approach of ethylene and jasmonate is mainly dependent on the interaction of their downstream signaling pathway [77]. Jasmonic acid encourages the synthesis of lycopene in tomato independently to ethylene and exogenously applied ethylene is widely used to trigger and initiate ripening in climacteric fruits [78,79]. Correspondingly, ethylene does not only affect the biochemical structure but also increases the respiration rate of fruit and vegetables [80]. Likewise, the exogenously applied melatonin influenced the ethylene biosynthesis pathway and conferred better aroma, color, sugar and overall postharvest quality of tomato [81]. The research provides a good base for utilizing melatonin in keeping the postharvest quality of produces. Both of these hormones regulated by melatonin play an important role in defining the postharvest status of produce by means of their possible involvement in providing resistance against postharvest diseases and deterioration. Still, not a great deal of research material is available on melatonin postharvest application. However, melatonin may be considered as a potential substance to reduce the percentage of postharvest losses and enhance the shelf life of postharvest produce. According to a recent report, the silencing of *fruit shelf-life regulator* (*SIFR*) gene has been reported for controlling the postharvest ripeness in tomato and also extended the fruit shelf life by inhibiting the ethylene production [82]. For that reason, it will be interesting to see how exogenous melatonin affects the postharvest maturity by regulating the expression level of *SIFR* gene. Furthermore, Table 2 represents the reported studies on melatonin application over postharvest products. 

## 5. Role of Melatonin in Mitigating Abiotic Stresses

In recent times, melatonin as a biostimulant and plant growth regulator attracts the interest of plant biologists [14]. For instance, it provides physiological and molecular resistance against many abiotic stresses by means of its involvement in regulating stress signaling [92,93]. Additionally, its beneficial effect on photosynthesis and other growth-related factors amongst different crops under the diverse abiotic stresses is another promising aspect of melatonin application [94,95]. Exogenous melatonin significantly induced the level of endogenous Abscisic acid (ABA) and Gibberellic acid (GA) in cucumber seedling under the saline condition, due to which the resistance against salinity was improved [96]. While in plants affected by heat stress, the level of cytokinin (CK) was degraded gradually. Though, induction in the level of CK biosynthesis was observed after the plants were treated with exogenous melatonin. The study further reported that the resistance against heat stress was perceived in the melatonin-treated plants due to enhanced CK level [97]. In short, the main action of mechanism is the improvement of the antioxidant defense system and enhancing photosynthetic activity (Table 3). Moreover, melatonin has been described by many scientists to significantly influence the overall plant growth against abiotic stresses [87,97,98,99] with minimum effects on the surrounding environment. 

## 6. Melatonin Role in Suppressing Biotic Stresses

To the best of our knowledge, the first study conducted on melatonin’s ability to increase resistance to biotic stress in plants was reported by Yin et al. [129]. In their study, they successfully used melatonin to mimic the harmful effects of *Diplocarpon mali* in apple tree through the root irrigation method. In another study, the *SNAT* mutant line in *Arabidopsis* suffered from avirulent pathogen *Pseudomonas syringae* pv due to the reduced induction capacity of defense genes (*PR1, ICS1*, and *PDF 1.2*). However, the induction was restored with the application of exogenous melatonin, confirming the role of melatonin in suppressing biotic stress [20]. Similarly, in apple juice, the melatonin showed excellent anti-microbes activity by reducing the percentage to 19% compared with control [83]. Moreover, the application of exogenous melatonin on the *Arabidopsis* plant augmented the level of endogenous melatonin and nitric oxide (NO) that was lethal against the pathogen *Pseudomonas syringe* pv. tomato (Pst) DC3000 [24]. So far it is known that melatonin expresses the activity of chitinase genes [129], which is an important factor in restricting the lesion expansion and inhibiting the growth of pathogen [69]. However, the induction in endogenous melatonin activity also plays an important role in sustaining the defense system [24]. More generally, that melatonin induces resistance against biotic stress is a collective action of endogenous hormonal [20], antioxidant enzymes activities and expression of *PR* and *Chitinase* genes. 

Furthermore, the melatonin-directed regulation of other phytohormones could provide resistance against biotic stresses, which is yet to be examined in plant [64]. For example, ethylene enhances the infection capacity and symptoms development as observed for cucumber inoculated with *Cucumber mosaic virus* (*CMV*) [130,131]. However, treatment with exogenous melatonin suppresses the ethylene activity [73] and could help in sustaining the plant defense system against that particular virus. A similar case is with other phytohormones as they are highly involved in keeping the balance of the plant defense system [63,132,133] but virus infection inanimates the plant hormones system, which helps them to replicate quickly [134]. Therefore, it could be of great importance to unravel the role of melatonin mediated defense response against plant viruses and other biotic stress factors. The reported studies in Table 4 demonstrated the potential role of melatonin application against biotic stress. However, more research is needed on the melatonin application against plant viruses, nematodes, and insects.

## 7. Regulation of Gene Expression

Gene expression is defined as a biological process which changes according to environmental stimuli. Sometimes these environmental stimuli induce positive and negative gene expression which further delimits the biological processes and production capacity of plants. Recently, various biological gene regulators have been reported [69,137,138]. Here we discussed the melatonin role in regulating and expressing gene activities in different plants under various stress conditions. Additionally, an *Arabidopsis* mutant, *SNAT*, was subjected to cold stress, whose anthocyanin producing ability was quite low compared to that of wild-type. However, after the application of exogenous melatonin, a restoration process was upheld due to the up-regulation of anthocyanin biosynthesis genes, which confirmed that the melatonin improves plant growth via the induction of anthocyanin activities [27]. In another report, cucumber seeds primed with melatonin have been employed to the RNA-seq approach. The study reported that 121 and 196 genes were up and down-regulated respectively. Among them, genes responsible for carbohydrate metabolism, cell wall synthesis, and lateral root formation were reported for exhibiting mix expression pattern [139]. The study made inroads for further functional characterization of the genes involved in lateral root formation, other biological processes and highlighted the all-important role of melatonin involvement in gene expression and regulation. Besides that, a stability analysis of reference genes was performed for the anti-cancerous medicinal plant *Catharanthus roseus* under exogenous melatonin treatment. The study confirmed that *EXP* and *EXPR* were the most stable genes under melatonin treatment, which is important for the future research in order to achieve an accurate expression pattern [140]. Melatonin is also involved in regulating stress-specific genes. For example, the *Arabidopsis* plant under iron deficient condition restored its tolerance to iron deficiency by regulating *FIT1, FRO2*, and *IRT1* genes after melatonin treatment [141]. The study confirmed that melatonin can increase the tolerance of the plant to iron deficiency by upregulating the iron stress-specific genes. While in Apple, the exogenously applied melatonin delayed leaf senescence by suppressing the chlorophyll degradation genes namely *senescence-associated gene 12 (SAG12)* and *AUXIN RESISTANT 3 (AXR3)/INDOLE-3-ACETIC ACID INDUCIBLE 17 (IAA17)* [122]. The same mechanism could be used for other stresses as well, though no study has stated it so far. In another report, the melatonin significantly upregulated the expression of *CmSOD*, *CmPOD*, and *CmCAT* and *MYB*, *bHLH*, *WD40* genes in melon and cabbage under cold and oxidative stress respectively [27,142]. The restoration of antioxidant enzymes activity is at the center of this induced resistance by these several expressed genes reported in Table 5. Moreover, there is a significant amount of study available on melatonin-induced gene expression, however, no study has examined the role of melatonin in regulating the *MLO clade* V genes, which is responsible for bringing susceptibility to powdery and downy mildew disease in different plants [143,144]. It could be worth studying the effect of melatonin on these important economic fungal diseases and also the genes responsible for their induction, as melatonin does show the potential of inducing resistance against fungal diseases [14,136]. Furthermore, gene expression in different plant species regulated by melatonin is listed in Table 5.

## 8. Melatonin Defense Mechanism

The defense mechanism of melatonin continues to puzzle researchers as no definite defense mechanism has been proposed so far. Nevertheless, there are many suggested mechanisms of action. For example, the ability to scavenge H_2_O_2_ and the induction of antioxidant enzymes activities by melatonin helps to recover plants from abiotic stresses [112,116,147]. In another report, the melatonin was proposed to up-regulate the expression of heat shock protein (HSP) to mitigate the high-temperature stress [92]. While for biotic stress, the melatonin was anticipated for activating the NO and salicylic acid (SA) mediated defense signaling pathway by expressing the PR-protein (pathogenesis-related protein) immediately [24,148]. Mitochondria are the main powerhouse for energy production through aerobic respiration and play a key role in plant growth and development [149]. Additionally, mitochondria and chloroplast are referred to as the original site of melatonin synthesis in plants [28]. In another study, mitochondria were pinpointed as a major generation site for NO and ROS [150,151] and could be important in playing a key role in mitigating various stresses via NO accumulation and ROS regulation [151,152,153]. Though, the mitochondria can be damaged due to the over-production of ROS under environmental stresses [151]. However, melatonin was reported to recover the damaged mitochondria [154]. Therefore, from the recent research reported, we have proposed a new model of melatonin defense mechanism (Figure 1). 

## 9. Approaches to Inducing Endogenous Melatonin Level

### 9.1. Transgenic Approaches to Inducing Endogenous Melatonin Level

The enzymes responsible for regulating the melatonin biosynthesis pathway has been successfully overexpressed in various crops and showed good results by boosting the level of endogenous melatonin. These enzymes include tryptophan decarboxylase (TDC), tryptamine5-hydroxylase, arylalkylamine *N*-acetyltransferase (AANAT)/serotonin *N*-acetyltransferase, and *N*-acetylserotonin methyltransferase/hydroxyindole-*O*-methyltransferase (HIOMT) [11,26,155,156,157,158,159,160]. In line with this, Apple *MZASMT1* overexpression in *Arabidopsis* increased the production of melatonin and enhanced the resistance against drought stress by lowering the activity of ROS [161]. In another report, the tomato lines overexpressing the *N*-acetylserotonin methyltransferase (*ASMT*) gene improved the level of endogenous melatonin. Additionally, it increased the cellular protection through the generation of heat shock protein (HSP) and activation of autophagy to refold denatured proteins that triggered heat resistance [92]. Moreover, the rice chloroplast caffeic acid *O*-methyltransferase COMT transgenic lines produced a higher level of melatonin by regulating the 5-methoxytryptamine (5-MT) pathway, which further resulted in improved seedling growth under continuous lighted environment [162]. Similarly, in tomato, the upregulation in *COMT1* transcription factor occurred in the plants due to the overexpression of the *HsfA1a* gene. Additionally, the induction of melatonin biosynthesis and resistance against cadmium stress was also observed in the overexpressed plants [163]. The overexpression of tryptophan decarboxylase 2 (MeTDC2)-interacting proteins, *N*-acetylserotonin *O*-methyltransferase 2 (*MeASMT2*) interacting proteins, and *N*-acetylserotonin *O*-methyltransferase 3 (*MeASMT3*) produced more melatonin than any other enzyme in cassava protoplast. Furthermore, the co-overexpression of these three enzymes with MeWRKY20/75 activated the MeWRKY20 and MeWRKY75 W-box transcriptional activities along with the positive effect on endogenous melatonin level [36]. Taken together, these findings suggested that the transgenic induction of the endogenous melatonin level could be a useful and reliable approach to understand the mechanism of this important signaling molecule in a plant system.

### 9.2. Some Other Strategies to Induce Endogenous Melatonin Level

Apart from the transgenic approach, there are other ways to induce the endogenous melatonin level in plants. An *Arabidopsis* melatonin mutant was unable to tackle the avirulent pathogen due to the decrease in melatonin level. However, exogenous melatonin recovers the plant defense mechanism and restores resistance against a pathogen by recovering the endogenous melatonin level [20]. In another study, the endophytic bacterium *Pseudomonas fluorescens* RG11 strain was used successfully to induce endogenous melatonin level in grapes. The inoculated grape plants showed resistance to salt stress by decreasing the reactive oxygen species burst and cell damage [164]. Similarly, the *Bacillus amyloliquefaciens* SB-9 endophytic bacterium strain from grapevine root promotes the endogenous melatonin production and also ameliorates the adverse effect of salt and drought stress via scavenging H_2_O_2_ activities [165]. Altogether, these environmentally friendly approaches of inducing endogenous melatonin can be utilized against biotic and abiotic stresses in agronomic and horticultural crops.

## 10. Conclusions

The plethora of research available about melatonin proves that it is an indispensable signaling molecule. The plant produces melatonin endogenously and research showed that it is highly important in maintaining plant growth and development. Additionally, the mitigation of abiotic and biotic stresses makes it a more versatile molecule. Moreover, it significantly reduces the percentage of losses during postharvest storage among different fruits and vegetables. Furthermore, the regulation of gene expression and crosstalk with other phytohormones is another important factor of melatonin, which contributes greatly to many plant biological processes under both normal and unfriendly environmental conditions. However, the endogenously produced melatonin sometimes is not enough to tackle harsh scenarios. For that reason, exogenous melatonin and some other approaches were implemented to induce the level of endogenous melatonin in order to sustain plant immunity and normal growth capacity. In addition, melatonin is considered as a nontoxic biodegradable molecule, which could be used for the promotion of organic farming [166]. To sum up, melatonin showed great importance across different plant science sectors; however, there is still no evidence available regarding the use of melatonin against viruses, nematodes, or insects; this requires further investigation.

## Figures and Tables

**Table 1:** Reported concentration of melatonin in some edible plants.

Crop	Used Methodology	Melatonin Content (pg/g FW(DW) Tissue)	Reference
Apple	GC-MS	0.16	[40]
Asparagus	RIA	9.5	[41]
Barley	LC	500–12,000 R; 82,300 S	[42,43]
Cucumber fruit seeds	RIA	24.6, 11,000	[41,44]
Chilies	UHPLC-MS/MS	31–93	[45]
Kiwi	RIA	0.02	[41]
Kidney bean	ELISA	529 DW	[46]
Rice	HPLC	100 L; 500 S; 200 R; 400 Fl	[47,48]
Sunflower	HPLC	29,000 DW	[16]
Tea (Shiya green tea)	HPLC	2.12 μg g^−1^	[49]
Tomato	LC	15,000–142,000 L	[50]
Wheat	LC	124,700 S	[42]

Abbreviation: L = leaf, R = roots, FL = flower, GC-MS = gas chromatographic-mass spectroscopy, RIA = radioimmunoassay, LC = liquid chromatography, UHPLC-MS/MS = Ultra-high performance liquid chromatography coupled to mass spectrometry in tandem mode, ELISA = Enzyme-linked immunosorbent assay, HPLC = High performance liquid chromatography.

**Table 2:** Effect of melatonin on postharvest produce.

Crop	Stress/Condition	Concentration	Functional Improvement	Reference
Apple	Browning	250 mg/L	Prevented apple juice from browning	[83]
Banana	Quality improvement	50–500 μM	Slowed down ripening, low ethylene production, accelerate endogenous melatonin.	[84]
Broccoli	senescence	100 μM/L	Maintained postharvest freshness	[85]
Cabbage	Cold	100 μM/L	Enhanced anthocyanin activity and antioxidant capacities	[27]
Cassava	Hydrogen peroxide	500 mg/L	Delayed postharvest physiological and root deterioration in cassava	[74,86]
Cucumber	Cold	500 μM	Increased protection against cold-induced oxidative stress in seeds	[87]
Peach	Oxidative	0.1 mmol/L	Slow down the senescence, increased antioxidant enzymatic activities and ascorbic acid content	[70,88]
Pear	Quality improvement	100 μM	Slowed senescence process, increase antioxidants, less fruit firmness losses, exhibited to be a strong scavenger of ROS	[89]
Strawberry	Fungal, quality improvement	1000 μmol/L or 100 μmol/L	triggered H_2_O_2_ accumulation, higher SOD activity, delayed senescence, decay, weight losses, maintained fruit firmness, titratable acidity, increased total phenol, flavonoids and antioxidant activity	[90,91]
Tomato	Quality improvement trial	50 μM	Promotes ripening, upregulated the expression level of fruit color development genes and altered the ethylene production.	[81]

**Table 3:** Protective role of melatonin in various crops against different abiotic stresses.

Crop	Stress Condition	Concentration	Functions	Reference
*Arabidopsis*	Heat	1000 μM	Improved seed germination under heat stress	[100]
Apple	Drought	100 µM	Reduced ABA activity and radical scavenging	[101]
Apple	Waterlogging	200 μM	Reduced chlorosis and wilting of the seedlings	[102]
Barley	Senescence	1 mM	Boosted chlorophyll content	[103]
*Brassica napus* L.	Drought	0.05 mmol/L	Increased the overall growth indices of brassica seedlings	[104]
Bermuda grass	Cold	100 μM	Induced photosynthetic activity under cold stress	[105]
Cucumber	Salinity	100 μM	Overall growth	[95]
Cucumber	Cinnamic acid	10 μM	Rescued cucumber seedlings from Cinnamic acid stress and increased the allocation of dry weight in roots.	[106]
Eggplant	Cadmium stress	150 μmol/L	Enriched photosynthetic activity	[107]
Faba bean	Salinity	500 μM	Enriched photosynthetic activity and mineral accumulation	[108]
Grapes	Water deficient	200 μmol/L	Amended antioxidative enzymes activity	[94]
Maize	Drought	100 μmol/L	Photosynthesis and growth	[109]
Melon	Cold	200 μM	Improved proline and ascorbic acid content	[110]
*Medicago sativa*	Drought	10 μM	regulation of nitro-oxidative and osmoprotective homeostasis	[111]
*Malus hupehensis*	Salinity	0.1 mM	Improved photosynthetic activity and better plant growth	[112]
*Malus hupehensis*	Alkaline	5 µM	Significantly induced the tolerance against alkaline stress by increasing the antioxidant activity and biosynthesis of polyamines	[113]
Perennial ryegrass	High temperature	20 μM	Regulate abscisic acid and cytokinin biosynthesis	[97]
Potato	Salinity	100 µM	Better chlorophyll content, antioxidant activities and water content	[114]
*Pisum sativum* L.	Oxidative stress	50 μM	Reduced O_2_^•^^−^ accumulation in leaf tissues and preservation of photosynthetic pigments	[115]
Rice	Salinity	20 μM	Delay leaf senescence and cell death in rice	[116]
Red cabbage	Heavy metal	10 μM	Improved seed germination and reduced the toxic effect of metal on the seedling.	[117]
Soybean	Multiple stress	100 µM	Boost and maintain the overall plant growth	[54]
Soybean	Aluminum stress	50 μM	Enhanced root growth and reduced aluminum toxicity	[118]
Sunflower	Salt	15 μM	Regulate root growth and hypocotyl elongation under salt stress	[119]
Tomato	Cold and salinity	100 μM	Improved photosynthesis and regulation of photosynthetic electron transport	[120,121]
Tomato	Heat and salinity	100 μM	Induced antioxidant enzymes activity and better photosynthetic performance	[122]
Tomato	Acid rain	100 μM	Enhanced tolerance against simulated acid rain and increased the photosynthetic activity	[123]
Tea	Cold	100 μM	Triggered photosynthetic and antioxidant enzymes activities	[62]
Watermelon	Salinity	150 μM	Redox homeostasis and improved photosynthetic activity	[124]
Watermelon	Vanadium stress	0.1 μM	Lower the concentration of vanadium in leaf, stem and better photosynthetic and antioxidants activity	[125]
Watermelon	Cold	150 μM and 1.5 μM	Alleviate cold stress by inducing long-distance signaling in the untreated tissue.	[126]
Wheat	Drought and nano-ZnO	500 μM and 1 mM	Augmented seedling percentage, growth, and antioxidant enzymes activities.	[59,127]
Wheat	Cadmium stress	50 mM	Reduce the level of hydrogen peroxide which increases the wheat plants growth	[128]

**Table 4:** Defense mechanism induced by melatonin against biotic stresses in different plants.

Crop	Pathogen	Concentration	Beneficial Functions	Reference
Apple	*Diplocarpon mali*	0.1 mM	Improved resistance to apple blotch disease	[129]
*Arabidopsis*	*Pseudomonas syringae*	10 μM	Increased the resistance by suppressing the bacterium about 10-fold	[19]
*Arabidopsis*	*Pseudomonas syringae*	10 μM	Alternatively, increased the resistance by triggering the level of endogenous salicylic acid.	[20]
Banana	*Fusarium oxysporum*	100 μM	Induce resistance in banana against the pathogen attack	[135]
*Lupinus albus*	*Penicillium* spp.	70 μM	Enhanced resistance against the fungal pathogen	[14]
Potato	*Phytophthora infestans*	5 mM	Inhibited the potato late blight disease by arresting the mycelial growth	[136]
Strawberry	*Botrytis cinerea* and *Rhizopus stolonifer*	1000 μmol/L	Attenuating fungal decay and maintaining nutritional quality of strawberry fruits	[90]
Tobacco	*Pseudomonas syringae*	10 μM	Increased the resistance by suppressing the bacterium about 10-fold	[19]

**Table 5:** Role of exogenous melatonin in regulating gene expression.

Crop	Stress/Conditions	Genes	Expression	Functions	Reference
*Arabidopsis thaliana*	Iron deficiency	*FIT1, FRO2, IRT1*	↑	Increased plants tolerance to Fe deficiency	[141]
*Arabidopsis thaliana*	Oxidative	*AtAPX1, AtCATs*	↑	Removed damaged protein via the activation of autophagy	[145]
*Arabidopsis thaliana*	Heat	*HSFA2, HSA32*	↑	Activated thermotolerance related genes in quadruple knockout mutant	[23]
Apple	Oxidative stress	*MdTDC1, MdT5H4, MdAANAT2*, and *MdASMT1*	↓	Slowing the decline in chlorophyll concentrations, restraining membrane damage and lipid peroxidation	[146]
Cabbage	Oxidative	*MYB, bHLH, WD40*	↑	Enhanced anthocyanins accumulation and increased antioxidant activities.	[27]
Melon	Cold	*CmSOD*, *CmPOD*, and *CmCAT*	↑	Recovered melon from cold stress through regulation of antioxidant activities	[142]
Potato	Salinity	*SDP1, LACS6, LACS7*, and *ACX4*	↑	Maintenance of PM HC–ATPase activity and KC/NaC homeostasis and increase tolerance to salinity stress	[114]
Peach	Cold	*PpAPX1*, *PpAPX3*, *PpAPX4*, and *PpAPX7*	↑	Activated the expression of genes involved in ASA-GSH cycle which improved resistance to cold stress	[70]
Peony	Fluctuating light	*TDC*	↑↓	Controls the production of melatonin biosynthesis under changing light spectrum	[30]
Rice		*OsARF*, *OsSAUR*	↑	Regulate rice root architecture on auxin dependent signalling manner	[58]
Tomato	Salinity and Heat	*SlcAPX, SlGR1, SlGST*, and *SlPh-GPX*	↑	Enhanced tolerance to multiple stress by activating antioxidant enzymes system	[122]
Watermelon	Vanadium	*Cla018095, Cla009820, Cla012125*	↑	Improved chlorophyll content and antioxidant activities	[125]
Wheat	Drought	*APX* and *MDHAR4*	↑	Increase tolerance against drought stress	[127]

↑ showing up-regulation and ↓ down-regulation of the respective genes presented in the table.
